# An enhanced secretary bird optimization algorithm based on precise elimination mechanism and boundary control for numerical optimization and low-light image enhancement

**DOI:** 10.1371/journal.pone.0331746

**Published:** 2025-09-08

**Authors:** Yuqi Xiong

**Affiliations:** School of Computer Science and Engineering, Southeast University, China; Indiana University School of Medicine, UNITED STATES OF AMERICA

## Abstract

Metaheuristic optimization algorithms often face challenges such as complex modeling, limited adaptability, and a tendency to get trapped in local optima when solving complex optimization problems. To enhance algorithm performance, this paper proposes an enhanced Secretary Bird Optimization Algorithm (MESBOA) based on a precise elimination mechanism and boundary control. The algorithm integrates three key strategies: a precise population elimination strategy, which optimizes the population structure by eliminating individuals with low fitness and intelligently generating new ones; a lens imaging-based opposition learning strategy, which expands the exploration of the solution space through reflection and scaling to reduce the risk of local optima; and a boundary control strategy based on the best individual, which effectively constrains the search range to avoid inefficient searches and premature convergence. Experimental validation shows that on 23 benchmark functions and the CEC2022 test suite, MESBOA significantly outperforms the original Secretary Bird Optimization Algorithm (SBOA) and other comparative algorithms (such as GWO, WOA, PSO, etc.) in terms of convergence speed, solution accuracy, and stability. Taking low-light image enhancement as an application case, MESBOA performs better in metrics such as Mean Squared Error (MSE), Peak Signal-to-Noise Ratio (PSNR), and Structural Similarity Index (SSIM) by optimizing the parameters of the normalized incomplete Beta function, verifying its effectiveness in practical problems. The research indicates that MESBOA provides an efficient solution for complex optimization tasks and has the potential to be promoted and applied in multiple fields.

## 1. Introduction

In the modern digital era, images play a crucial role across various domains. In the field of computer vision, images serve as fundamental data for tasks such as object recognition and scene understanding [1]. In medical imaging, clear and accurate images are essential for disease diagnosis and treatment planning. In security surveillance, high-quality images facilitate the identification of suspicious behaviors and individuals. However, images captured in low-light environments often suffer from significant quality degradation, severely limiting their effectiveness in these applications. Low-light images commonly exhibit low contrast, which reduces the distinction between different objects, making it difficult to accurately identify target objects. Additionally, image details become blurred, obscuring critical information. For instance, in nighttime surveillance images, essential details such as facial features and license plate numbers may not be clearly visible [2,3]. Moreover, low-light conditions contribute to a reduced signal-to-noise ratio (SNR), where noise interference further degrades image quality, making it more challenging to interpret and analyze. These issues not only pose significant challenges for subsequent image processing but also limit the practical applications of related technologies in real-world scenarios.

Traditional low-light image enhancement methods can be broadly categorized into histogram-based methods and Retinex theory-based approaches [4]. Histogram-based methods, such as histogram equalization (HE), enhance global contrast by redistributing pixel intensity values. However, these methods often lead to issues such as local over-enhancement, detail loss, and unnatural visual effects [5]. Adaptive histogram equalization (AHE) improves adaptability to local brightness variations but tends to amplify noise. Contrast-limited adaptive histogram equalization (CLAHE) mitigates excessive noise amplification but performs suboptimally in enhancing complex low-light images [6]. Retinex theory-based methods [7], including single-scale and multi-scale Retinex algorithms [8,9], enhance brightness and contrast by decomposing an image into reflectance and illumination components and adjusting the illumination component accordingly. However, in practical applications, these methods require careful parameter tuning, and their performance is highly influenced by image scene complexity. The optimal parameter selection varies significantly across different scenes, increasing the difficulty of practical implementation.

Yin et al. proposed an image enhancement algorithm based on weight-constrained decision-making. By constructing a gray histogram equalization model and assigning fusion weight factors to the image, the algorithm achieves enhancement effects [10]. Pashaei et al. proposed a novel quantum behavior arithmetic optimization algorithm (QAOA) for medical image processing, which utilizes a mutation operator with a Gaussian probability distribution as a powerful strategy to enhance the performance of QAOA and prevent premature convergence to local optima. The Gaussian QAOA (GQAOA) is specifically tailored for medical image enhancement and is combined with Contrast Limited Adaptive Histogram Equalization (CLAHE) to improve the informational content and details of medical images [11]. Anil Kumar et al. introduced a dark image enhancement method that integrates swarm intelligence optimization with segmented gamma correction, leveraging the advantages of gamma correction and histogram equalization [12]. Abhishck et al. explored the enhancement of the raw ISIC2017 skin cancer dataset using a GAN-based approach [13].To improve the quality of image dehazing, Bu Ning et al. proposed a novel dehazing model based on fractional-order derivatives and a data-driven regularization term [14]. To address the inefficiency in parameter selection for IBF, the limited variable parameter range for stretching high or low grayscale areas, and the near-inactivity of image enhancement at both ends of the stretch, Braik et al. proposed a hybrid Whale Optimization Algorithm (WOA) with a Chameleon Swarm Algorithm (CSA), referred to as HWOA, for adaptively determining the optimal parameters of IBF for ICE. They then applied bilateral gamma correction (BGC) to produce better contrast and brightness while preserving edge details [15]. Additionally, Pashaei et al. introduced a novel hybrid method called PSOKHA, which combines the Krill Herd Algorithm (KHA) and Particle Swarm Optimization (PSO) for image enhancement. The proposed PSOKHA method was used to search for the optimal transfer function parameters to improve image quality [16]. Lqbal et al. suggested the use of MI-GAN-based image enhancement techniques to generate retinal images [17]. Jiang et al. combined traditional image feature fusion techniques with convolutional sparse coding and image enhancement methods [18]. Additionally, Tubbs introduced a normalized nonlinear Beta function to fit nonlinear transformation curves [19]. In the field of image enhancement, the nonlinear Beta transformation has demonstrated excellent performance. However, its effectiveness depends on parameter tuning. Traditional parameter adjustment methods for the nonlinear Beta function primarily rely on manual selection and exhaustive search, which lack efficiency and adaptability [20,21]. In contrast, metaheuristic optimization algorithms provide an effective tool for addressing this issue, as they excel in exploring large-scale solution spaces to determine optimal parameters.

In recent years, with the rapid advancement of artificial intelligence, metaheuristic optimization algorithms have demonstrated unique advantages in low-light image enhancement. These algorithms, inspired by natural phenomena or biological behaviors, explore vast solution spaces to identify optimal solutions.

For instance, the Genetic Algorithm (GA) [22]. mimics the process of natural selection and evolutionary adaptation, iteratively evolving a population of candidate solutions to converge towards the optimal solution. Differential Evolution (DE) [23] leverages the principles of natural selection and reproduction, incorporating stochastic operators such as selection, crossover, and mutation. Particle Swarm Optimization (PSO) [24] is inspired by the social behavior of birds and fish, where particles adjust their movement based on their own experiences and those of their neighbors to search for optimal solutions. Several other bio-inspired algorithms have been developed based on the intelligent behaviors of various species. The Ant Colony Optimization (ACO) [25] is derived from the foraging behavior of ants, which use pheromone trails to identify the shortest path between their nest and food sources. The Grey Wolf Optimizer (GWO) [26] models the social hierarchy and hunting strategies of grey wolves. The Artificial Gorilla Troops Optimizer (GTO) [27] is inspired by the social behavior of gorillas. The Grasshopper Optimization Algorithm (GOA) [28] simulates the life patterns of grasshoppers, where the local exploration phase corresponds to short-range movements of larvae, while the global search phase mimics the long-range migration of adult grasshoppers, following a coordinated search strategy.

Other algorithms include the Whale Optimization Algorithm (WOA) [29], inspired by the hunting behavior of whales, and the Bat Algorithm (BA) [30], which mimics the echolocation strategy of bats. The Nutcracker Optimizer (NOA) [31] is based on the nut-cracking and cache-searching behaviors of Clark’s nutcracker. The Starling Murmuration Optimizer (SMO) [32] simulates the astonishing murmuration behavior of starlings. The Crayfish Optimization Algorithm (COA) [33] is developed based on the foraging, competition, and heat-avoidance behaviors of crayfish. Similarly, the Coyote Optimization Algorithm (COA) [34] models the social organization and survival strategies of North American coyotes. The Dung Beetle Optimizer (DBO) [35] is inspired by the rolling, foraging, stealing, and reproductive behaviors of dung beetles. The Golden Eagle Optimizer (GEO) [36] mimics the spiral trajectory adjustments of golden eagles during hunting. The Salp Swarm Algorithm (SSA) [37] is based on the swarming behavior of salp in the ocean. Furthermore, some optimization algorithms are inspired by human activities. The Gold Rush Optimizer (GRO) [38] models the trial-and-error process of gold prospectors searching for gold deposits in riverbeds. The Secretary Bird Optimization Algorithm (SBOA) [39] is based on the survival behavior of secretary birds. The Chimpanzee Optimization Algorithm (ChOA) [40] simulates the coordinated attack, chase, interception, and deterrence strategies of chimpanzees. The Artificial Rabbits Optimization (ARO) [41] is designed based on the survival strategies of rabbits, including evasive foraging and random hiding.

These algorithms have demonstrated their ability to autonomously search for optimal parameters in image enhancement, often yielding superior results compared to traditional methods. For instance, Han et al. [42] proposed a whale optimization-based image enhancement method using transformation functions to enhance both global and local details in grayscale images. Du et al. [43] addressed the inefficiencies of the traditional incomplete Beta function in image enhancement—particularly its limited parameter adjustability in low- and high-gray regions and ineffective contrast enhancement in mid-range gray levels. They introduced a method combining bilateral gamma correction with the incomplete Beta function, where the chimpanzee optimization algorithm (ChOA) was used to adaptively select the function’s parameters, leading to improved contrast in color images. Veluchamy et al. [44] proposed a weighted gamma correction method optimized by the Artificial Bee Colony (ABC) algorithm to enhance contrast-distorted images. By improving contrast and brightness, their approach produced visually superior images with enhanced fine details. To address the limitations of conventional denoising methods in handling high-noise images, Huang et al. [45] developed an image denoising algorithm based on an improved whale optimization algorithm and parameter-adaptive stochastic resonance. Similarly, Qu et al. [46] introduced a medical image denoising method using an improved Black Widow Algorithm (BWA), which enhances the quality of medical images and improves diagnostic accuracy. Although these metaheuristic-based image enhancement methods have significantly improved image quality, they still lack intelligent adaptability, require complex modeling processes, and pose challenges for widespread application. Future research should focus on integrating deep learning techniques with metaheuristic algorithms to achieve more adaptive and efficient image enhancement solutions.

Secretary Bird Optimization Algorithm (SBOA) [39], as an emerging metaheuristic algorithm, is inspired by the hunting and escape behaviors of the secretary bird. SBOA has shown potential in solving certain optimization problems. However, the algorithm also exhibits some inherent drawbacks. In the early stages of optimization, its convergence speed is relatively slow, requiring more time and computational resources to obtain a reasonably good solution. Additionally, when dealing with complex problems, SBOA tends to get trapped in local optima, failing to find the global optimum, which adversely affects the overall performance of the algorithm. To address these issues, Yu Zhu et al. proposed a quantum computing-based, multi-strategy improved Secretary Bird Optimization Algorithm (SBOA). By introducing the concept of velocity and integrating the Particle Swarm Optimization (PSO) search mechanism, they provided a new search approach for SBOA, enhancing population diversity. They also introduced a quantum mutation strategy, utilizing quantum computing to interfere with the mutation factor at the optimal position, which improves the algorithm’s local exploration capability [47]. Furthermore, another group proposed a multi-strategy enhanced SBOA by incorporating an incremental PID control feedback mechanism, a golden sine guidance strategy, and a cosine similarity update strategy. This approach improved the algorithm’s solution accuracy and search ability [48]. While these modifications have improved SBOA, some inherent flaws still remain.

To address these issues, this paper proposes a Multi-Strategy Enhanced Secretary Bird Optimization Algorithm (MESBOA). The algorithm integrates three improvement strategies: First, it introduces a precise population elimination strategy, which eliminates individuals with poor fitness and generates new individuals in a more intelligent manner. This optimizes the population structure and increases the probability of obtaining high-quality solutions. Second, a lens-based reverse learning population update strategy is incorporated, which enhances the exploration capability of the solution space through reflection and scaling, thereby reducing the risk of the algorithm getting stuck in local optima. Finally, a boundary control strategy based on the best individual is introduced to effectively control the search space, avoiding inefficient searches and premature convergence.

The main contributions of this paper are as follows:

(1) Algorithmic Innovation: This study proposes MESBOA, an improved version of the Secretary Bird Optimization Algorithm (SBOA), by integrating a precise population elimination strategy, lens imaging-based opposition learning, and a boundary control strategy guided by the best individual. These enhancements effectively address the issues of slow convergence and susceptibility to local optima in SBOA, significantly boosting the algorithm’s overall performance.
(2) Rigorous Performance Validation: MESBOA was benchmarked against eight state-of-the-art algorithms across 23 standard test functions. The results demonstrate that MESBOA exhibits faster convergence, higher accuracy, and greater stability, thereby validating the effectiveness of the proposed enhancement strategies.
(3) Extension to Low-Light Image Enhancement: MESBOA was further applied to the task of low-light image enhancement, where it was used to optimize parameters based on the normalized incomplete Beta function. Experimental results show that MESBOA-enhanced images achieve superior visual quality and outperform existing methods across various evaluation metrics.

The remainder of this paper is structured as follows: Section 2 introduces the fundamental principles of SBOA and provides a detailed explanation of the proposed MESBOA. Section 3 presents experimental studies on MESBOA, including performance evaluation on benchmark functions, stability analysis, and computational cost analysis. Section 4 applies MESBOA to low-light image enhancement, describing the mathematical model of the image enhancement method and discussing the experimental results. Finally, Section 5 summarizes the research findings and outlines future research directions.

## 2. Secretary bird optimization algorithm and proposed method

This chapter primarily introduces the inspiration, mathematical model, and enhancements of the Secretary Bird Optimization Algorithm (SBOA), along with the proposed Multi-Strategy Enhanced Secretary Bird Optimization Algorithm (MESBOA). Inspired by the hunting and evasion behavior of secretary birds, SBOA constructs a mathematical model to perform optimization tasks by simulating these natural behaviors. However, to address the limitations of SBOA—such as slow convergence and a tendency to fall into local optima—MESBOA incorporates several improvement strategies. These include a precise population elimination strategy, a population updating mechanism based on lens imaging-based opposition learning, and a boundary control strategy guided by the best individual. The chapter provides a detailed explanation of the principles and implementation of these strategies, along with the pseudocode and computational complexity analysis of MESBOA.

### 2.1. Secretary bird optimization algorithm

#### 2.1.1. Inspiration.

The Secretary Bird Optimization Algorithm (SBOA) is inspired by the natural survival behaviors of the secretary bird, primarily simulating its predation strategies and escape mechanisms from predators or threats. The three-stage predation behavior of the secretary bird corresponds to the exploration phase of the algorithm, while its two escape strategies from predators and threats correspond to the exploitation phase of the algorithm [49,50].

#### 2.1.2. Mathematical model.

The SBOA is a population-based metaheuristic method, where secretary birds are considered members of the algorithm’s population. The position of each secretary bird in the search space determines the values of decision variables. Therefore, in SBOA, the position of a secretary bird represents a candidate solution to the problem. At the initial stage of SBOA implementation, the positions of secretary birds in the search space are randomly initialized using Equation (1):


$$\begin{array}{c} X=lb+rand\times (ub-lb) \end{array}$$ (1)

Where X represents the corresponding value of a secretary bird, ub and lb denote the upper and lower bounds of the problem, respectively, and rand is a random number within the range [0,1].

The hunting strategy and the escape strategy when encountering predators are the two natural behaviors that inspire the design of the SBOA. Therefore, in each iteration, every member of the secretary bird population is updated in two distinct phases.

**Exploration Phase (Hunting Strategy of the Secretary Bird):** The hunting behavior of the secretary bird when preying on snakes is typically divided into three stages: searching for prey, exhausting the prey, and attacking the prey. Based on biological statistics regarding the sequential timing and duration of each stage, the entire hunting process is divided into three equal phases: t<1/3 T、1/3T<t<2/3T and 2/3T<t<T, corresponding to the three stages of the secretary birds’ predation process. This process is mathematically modeled using Equation (2):


$$\begin{array}{c} {\;X}_{1}(t+1)=\left\{ \begin{array}{c} {\;X}_{i}(t)+R_{1}\times {(X}_{r_{1}}(t)-X_{r_{2}}(t)),\;if\;t<\frac{1}{3}T\\ \;\;\;\;\;\;\;\;\;\;\;\;X_{best}(t)+exp\left({\left(\frac{t}{T}\right)}^{4}\right)\times (RB-0.5)\times \left(X_{best}(t)-X_{i,j}(t)\right),\;if\;\frac{1}{3}T<t<\frac{2}{3}T\\ {\;X}_{best}(t)+{\left(1-\frac{t}{T}\right)}^{\left(2\times \frac{t}{T}\right)}\times X_{i,j}(t)\times RL,\;else \end{array}\right.\; \end{array}$$ (2)

where t represents the current iteration number, and T is the maximum number of iterations. ${\;X}_{1}(t+1)$ denotes the new state of the secretary bird in the first phase. and are randomly selected candidate solutions in the first-stage iteration. is an array of size 1×dim, randomly generated from the interval [0,1]. RB is an array of size 1×dim, randomly sampled from a standard normal distribution (mean = 0, standard deviation = 1). $x_{best}$ represents the global best solution. RL represents the Lévy flight function, which is computed using Equation (3).


$$\begin{array}{c} \left\{ \begin{array}{c} RL=0.5\times Levy(dim)\\ Levy(dim)=0.01\times \frac{u\times \sigma }{|v{|}^{\frac{1}{\eta }}}\\ \sigma ={\left(\frac{\Gamma (1+\eta )\times sin\left(\frac{\pi \times \eta }{2}\right)}{\Gamma \left(\frac{1+\eta }{2}\right)\times \eta \times 2\left(\frac{\eta -1}{2}\right)}\right)}^{\frac{1}{\eta }} \end{array}\right.\; \end{array}$$ (3)

In this equation, η is a fixed constant with a value of 1.5. u and v are random numbers uniformly distributed in the range [0, 1].Γrepresents the Gamma function, The value of η is also set to 1.5.

**Exploitation** **Phase (Secretary Bird Escape Strategy):** Secretary birds may encounter attacks from predators or competition for food. Being highly intelligent, they typically employ evasive strategies to protect themselves or their food. These strategies can be categorized into two types:

(1) Flight or Running Escape $\left(S_{1}\right)$ – The bird attempts to flee by running or flying.
(2) Camouflage Escape $\left(S_{2}\right)$ – The bird blends into the environment using colors or structures to avoid detection by predators.

This process is modeled using Equation (4):


$$\begin{array}{c} {\text{\textbackslash{} }X}_{\text{2}}(t+\text{1})=\left\{ \begin{array}{c} \;\;\;{S_{\text{1}}:\;X}_{best}(t)+(\text{2}\times RB-\text{1})\times {\left(\text{1}-\frac{t}{T}\right)}^{\text{2}}\times X(t)\text{, }\;\;\;\;if\text{\textbackslash{} \textbackslash{} }Q<R_{\text{2}}\\ S_{\text{2}}:{\text{\textbackslash{} \textbackslash{} }X}_{i\text{,}j}(t)+R_{\text{3}}\times \left(X_{rand}(t)-l\times X_{i\text{,}j}(t)\right)\text{, \textbackslash{} }\;\;\;\;\;\;\;\;\;\;\;else \end{array}\right.\; \end{array}$$ (4)

where Q=0.5, $R_{2}$ and $R_{3}$ are 1×dim arrays randomly generated from a normal distribution. $X_{rand}$ represents a randomly selected candidate solution in the current iteration, l is a randomly chosen integer with a value of 1 or 2.

During the Exploration and Development phases, if the objective function value improves at the new position, the secretary bird accepts the new position; otherwise, it retains its current position. This type of update is called an effective update, preventing the algorithm from moving into non-optimal regions. The process is modeled using Equation (5):


$$\begin{array}{c} X(t+1)=\left\{ \begin{array}{c} \;\;X_{i}(t+1),\;\;\;if\;f\left(X_{i}(t+1)\right)<f\left(X(t)\right)\\ X_{i}(t),\;\;\;\;\;\;\;\;if\;f\left(X_{i}(t+1)\right)\geq f\left(X(t)\right) \end{array}\right.\; \end{array}$$ (5)

Where, represents the new position of the secretary bird at each phase. i denotes the phase index (i=1 for the exploration phase and i=1 for the development phase). f(·) represents the objective function, which evaluates the fitness of a solution.

### 2.2. Proposed method

#### 2.2.1. Precision population elimination strategy.

**Fig 1:**
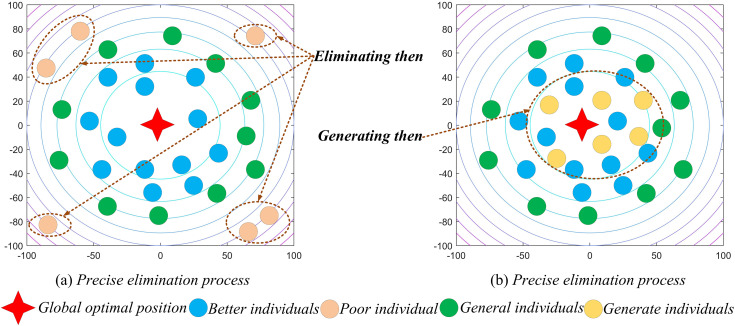
Precision elimination strategy.

To obtain high-quality solutions, many researchers have introduced reverse learning into algorithms. Although this strategy is effective, it also has certain drawbacks. For example, if the optimal solution happens to lie on the symmetric plane between the reverse learning solution and the original solution, the fitness values of the two corresponding solutions will be exactly the same. In this case, the strategy becomes ineffective. Therefore, this paper proposes a new strategy for obtaining high-quality solutions. Inspired by the evolutionary theory of “survival of the fittest,” a population precision elimination strategy based on maladaptive individuals is designed to optimize the population and increase the probability of obtaining better solutions. After one iteration, some of the individuals with the worst fitness are selected for elimination, and new individuals are generated to replace them based on the designed strategy, as shown in Equation (6).


$$\begin{array}{c} X(t+1)=X_{best}(t)+(ub-lb)\times R_{r}\times T\left(\frac{1}{m}\times exp{\left(\frac{ln\left(t_{c}\right)}{t_{c}}\right)}^{t}\right) \end{array}$$ (6)

here, ub and lb represent the upper and lower bounds of the search space, respectively, and m is the scaling factor, which is set to 10 in this study. $t_{c}$ is a critical point during the iteration process, and $R_{r}$ represents the dynamic radius defined by Equation (7):


$$\begin{array}{c} R_{r}=\text{1}-\frac{t}{T} \end{array}$$ (7)

where t represents the current iteration, and T represents the maximum number of iterations. By multiplying the mutation factor, dynamic radius, and search boundary range, which follow an adaptive t-distribution, the elastic radius t is constructed. This ensures that, even in the case of overall contraction, there remains a certain probability of expanding the range for generating new individuals, thereby preventing the clustering of new individuals in later stages. Consequently, the elastic radius determines the generation range of new individuals at different iteration stages, enabling large-scale exploration in the early stages and small-scale exploitation in the later stages. A schematic diagram of this population precision elimination strategy is shown in Fig 1.

#### 2.2.2. Population renewal strategy based on lens imaging reverse learning.

To address the slow convergence speed of SBOA in the early stages, this paper proposes a lens imaging reverse learning strategy. This strategy fully explores each dimensional component of the current solution and its reverse solution, combining the advantageous dimensions to generate a partially reflected reverse solution. This solution is applied to the leader individuals to generate new candidate individuals that can jump to more promising search areas, thereby enhancing population diversity and reducing the likelihood of the algorithm getting stuck in local optima. The lens imaging reverse learning strategy explores previously unexplored regions of the solution space by reflecting and scaling the solution, thereby increasing solution diversity and reducing the risk of the algorithm becoming trapped in local optima [51]. Furthermore, in the later stages of the algorithm, when the k-value is large, newly generated solutions become more concentrated around the current optimal solution. This concentration helps the algorithm fine-tune these solutions more precisely, accelerating convergence toward the global optimum or a solution close to the global optimum.

**Fig 2:**
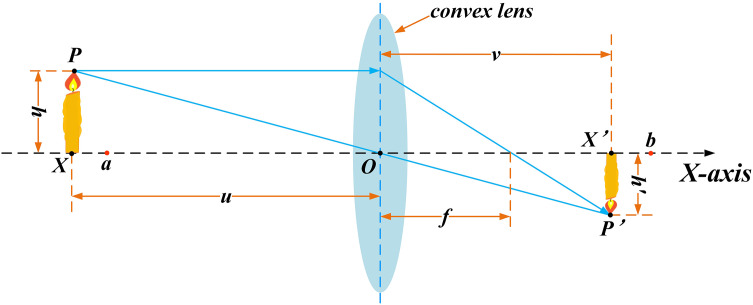
Lens imaging opposition-based learning.


As shown in Fig 2, suppose there is an individual P with a height h and its projection on the x-axis is X within the spatial range of the interval [lb, ub]. By using a convex lens placed at point O (where O is the midpoint of [lb, ub]) for imaging, the individual P, is transformed into a new individual $P^{\prime }$ with a height $h^{\prime }$ and a projection on the axis as $X^{\prime }$. The imaging principle is as follows:

$$\begin{array}{c} \frac{\frac{ub+lb}{2}-X}{X^{\prime }-\frac{ub+lb}{2}}=\frac{h}{h^{\prime }} \end{array}$$ (8)

Let $\frac{h}{h^{\prime }}=k$,and by rearranging the equation, we get:


$$\begin{array}{c} X^{\prime }=\frac{ub+lb}{2}+\frac{ub+lb}{2k}-\frac{X}{k} \end{array}$$ (9)

The scaling factor k is calculated as:


$$\begin{array}{c} k={\left(1+{\left(\frac{t}{T}\right)}^{2}\right)}^{10} \end{array}$$ (10)

This method enhances the exploration of the reverse solution space, significantly reducing the possibility of the algorithm getting trapped in local optima. It thoroughly explores and retains the advantageous dimensional information of both the current individual and the reflected reverse individual.

#### 2.2.3. Optimal individual-based boundary control strategy.

In optimization algorithms, boundary control plays a crucial role as it limits the range of the search space, preventing issues such as ineffective searches, premature convergence, and local optima. These problems can arise when the search process deviates from feasible solutions or exceeds the problem’s constraints. If the boundaries of the search space are set too narrowly, it may hinder the algorithm from fully exploring a broader solution space, leading to the algorithm becoming trapped in local optima. Therefore, to maximize the use of positional information from different individuals, a boundary control strategy based on the optimal individual $X_{best}$ is proposed. This strategy reflects individuals that exceed the boundaries back into the search space, ensuring that the population maintains a certain distribution throughout the feasible domain. This prevents excessive concentration of individuals near the boundaries, thereby increasing population diversity, reducing ineffective searches, and improving computational efficiency. This process is described by Equation (11).


$$\begin{array}{c} X=\left\{ \begin{array}{c} \frac{\left(lb+X_{best}\right)}{2},\;if\;X<lb\\ \frac{\left(ub+X_{best}\right)}{2},\;if\;X>ub \end{array}\right.\; \end{array}$$ (11)

The pseudocode for MESBOA is depicted as **Algorithm 1**.

**Algorithm 1. Pseudo-Code of MESBOA.**

*1: Initialize the secretary bird population* X *by* Equation (1)

*2: Initialization of the position of secretary bird and calculate the objective function*

*3:* ***while*** t=1:T ***do***

*4:  Update Secretary Bird* $X_{best}$

*5:  ****for*** i=1:N

*6:* ***Exploration phase (Hunting strategy):***

*7:   Calculate new status of secretary bird using* Equation (2)

*8:   Update the population member of secretary bird using* Equation (5) *and* Equation (8)

*9:   The individuals beyond the boundary are adjusted using* Equation (11)

*10:* ***EXploitation phase (Escape strategy):***

*11:   Calculate new status of secretary bird using* Equation (4)

*12:   Update the population member of secretary bird using* Equation (5) *and* Equation (8)

*13:   The individuals beyond the boundary are adjusted using* Equation (11)

*14:* ***end***

*15:  Generate new individuals using* Equation (6)

*16:  Update the best candidate solution*

*17:* t=t+1

*18:* ***end***

19: Output best candidate solution $\left(X_{best}\right)$

#### 2.2.4. Computational complexity analysis.

Analyzing the complexity of metaheuristic algorithms is crucial as it determines the time required for algorithm implementation. In the field of computer science, we typically employ “Big-O notation” to describe the time complexity and memory usage of algorithms. This paper conducts an in-depth complexity analysis using the “Big-O notation”. In the original SBOA algorithm, the complexity of random population initialization is O(N), while computing the fitness of each search agent has a complexity of O(N). The complexity of updating the current search agent’s position and fitness, as well as updating the global best solution’s position and fitness, is O(T×N)+O(T×N×Dim). Therefore, the overall complexity of the standard SBOA is O(N×(T×Dim + 1)). Since the MESBOA algorithm does not introduce new loop structures or additional update mechanisms, its complexity remains O(N×(T×Dim + 1)).

## 3. Experimental results

This chapter conducts a comprehensive performance evaluation of MESBOA through extensive comparative experiments. We rigorously test the algorithm using 23 challenging benchmark functions alongside the standardized CEC2022 test suite, comparing its performance against multiple state-of-the-art optimization methods. Our analysis systematically examines four critical aspects: convergence speed, solution accuracy, algorithm stability, and computational efficiency, providing a holistic assessment of MESBOA’s capabilities.

### 3.1. Test function and parameter Settings

This section evaluates the performance of the proposed MESBOA using 23 challenging benchmark functions [52] and CEC2022 test functions [53] from the Numerical Optimization Competition and compares it with other algorithms. The comparison algorithms include: Grey Wolf Optimizer (GWO) [26], Whale Optimization Algorithm (WOA) [29], Particle Swarm Optimization (PSO) [24], Differential Evolution (DE) [54], Dung Beetle Optimizer (DBO) [35], Crested Porcupine Optimizer (CPO) [55], Red-billed Blue Magpie Optimizer (RBMO) [56], Arctic Puffin Optimization (APO) [57], Black-winged Kite Algorithm (BKA) [58], Modified LSHADE-SPACMA with new mutation strategy and external archive mechanism(mLSHADE_SPACMA) [59] and Secretary Bird Optimization Algorithm(SBOA). The parameter settings for the algorithms are shown in Table 1. To ensure fairness and eliminate the effects of randomness, the same parameters are set for all algorithms: a population size of 30 and a maximum number of iterations of 500. Each algorithm is independently run 30 times, and the results are summarized in terms of average (Ave), standard deviation (Std), and ranking (Rank), with the best results highlighted in bold. All experiments are conducted in the following computational environment: Windows 10 operating system, hardware configuration of a 13th-generation Intel(R) Core(TM) i5-13400 processor (2.5GHz), 16GB RAM, and MATLAB 2024b as the software tool. This unified experimental setup and statistical method ensure the reliability and comparability of the results.

**Table 1:** Compare algorithm parameter settings.

Algorithms	Name of the parameter	Value of the parameter
GWO	a	[0,2]
WOA	r, l, a	[0,1], [-1,1], [0,2]
PSO	$c_{1},c_{2}$	1.5, 1.5
DE	F, Cr	0.8, 0.1
DBO	$P_{percent}$	0.2
CPO	$\alpha ,\;N_{min},\;T_{f},\;T$	0.1, 80, 0.5, 2
RBMO	r	0.9, [0,1]
APO	F, C	0.5, 0.5
BKA	P, r	0.9, [0,1]
mLSHADE_SPACMA	$|A|,\;P,H,\sigma ,L_{rate},\rho ,k$	1.4,0.11,5,0.5,0.8,0.11,3
SBOA	$l,\;R_{1},R_{2}$	{1,2}, [0,1], [0,1]
MESBOA	$l,\;R_{1},R_{2},R_{r}\;,\;$	{1,2}, [0,1], [0,1], [1,0]

### 3.2. Assessing performance with 23 benchmark functions

**Fig 3:**
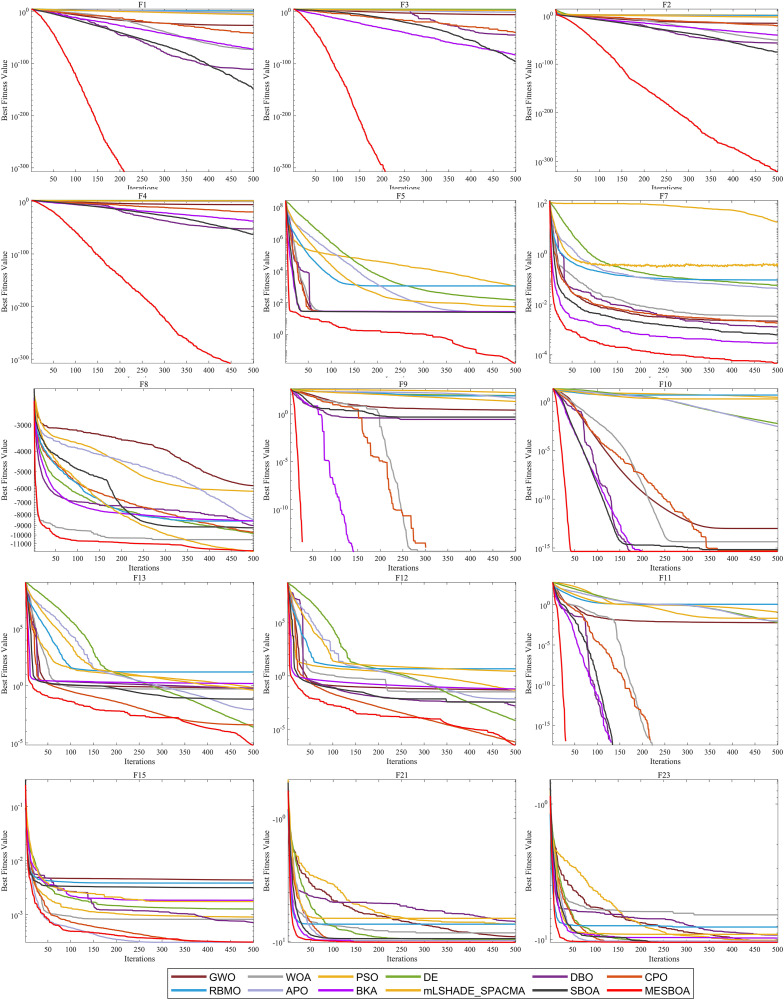
Convergence curves obtained by different algorithms on 23 benchmark functions.

In this subsection, the performance of MESBOA is evaluated using 23 benchmark functions. To comprehensively and thoroughly assess its performance, the experiments are conducted under a condition of 30 dimensions (dim = 30). The statistical results of the experiments are presented in Table 2. This table lists the mean (Ave), standard deviation (Std), and ranking (Rank) for each algorithm, with the best-performing results highlighted in bold. The convergence speed curves are shown in Fig 3.

**Table 2:** Results obtained by different algorithms on 23 benchmark functions.

F~	Metric	PSO	DE	GWO	WOA	COA	DBO	CPO	APO	BKA	mLSHADE_SPACMA	SBOA	MESBOA
F1	Ave	3.16E-27	1.42E-73	2.43E + 00	4.65E-04	1.37E-111	1.79E-42	2.92E + 01	1.95E-04	2.53E-73	7.52E-07	2.05E-148	**0.00E + 00**
	Std	7.19E-27	7.11E-73	9.59E-01	1.84E-04	7.48E-111	6.96E-42	1.02E + 02	2.62E-04	1.38E-72	9.75E-07	1.05E-147	**0.00E + 00**
	Rank	7	5	11	10	3	6	12	9	4	8	2	**1**
F2	Ave	1.12E-16	3.42E-51	4.45E + 00	2.21E-03	3.65E-57	2.29E-22	2.21E + 00	1.62E-03	3.08E-41	8.12E-04	4.21E-76	**0.00E + 00**
	Std	8.30E-17	1.11E-50	1.23E + 00	5.60E-04	1.96E-56	7.10E-22	3.99E + 00	8.32E-04	1.68E-40	2.08E-03	1.67E-75	**0.00E + 00**
	Rank	7	4	12	10	3	6	11	9	5	8	2	**1**
F3	Ave	2.73E-06	4.24E + 04	1.90E + 02	3.32E + 04	1.93E-45	2.34E-40	1.23E + 03	1.38E-01	9.72E-84	3.40E + 02	1.61E-96	**0.00E + 00**
	Std	4.88E-06	1.41E + 04	6.18E + 01	5.47E + 03	1.06E-44	1.00E-39	1.02E + 03	1.36E-01	5.32E-83	2.22E + 02	6.44E-96	**0.00E + 00**
	Rank	6	12	8	11	2	5	10	7	4	9	3	**1**
F4	Ave	7.09E-07	5.26E + 01	2.00E + 00	1.28E + 01	4.80E-53	1.19E-20	1.08E + 01	4.34E-01	3.10E-38	1.09E + 01	2.49E-63	**0.00E + 00**
	Std	6.57E-07	2.62E + 01	2.08E-01	2.26E + 00	1.88E-52	5.57E-20	3.59E + 00	2.08E-01	1.70E-37	2.70E + 00	1.33E-62	**0.00E + 00**
	Rank	6	12	8	11	3	5	10	7	4	9	2	**1**
F5	Ave	2.70E + 01	2.80E + 01	1.09E + 03	1.50E + 02	2.57E + 01	2.58E + 01	1.13E + 03	2.43E + 01	2.77E + 01	5.64E + 01	2.51E + 01	**1.63E-02**
	Std	8.10E-01	3.49E-01	7.57E + 02	5.20E + 01	1.77E-01	4.32E-01	1.33E + 03	8.94E + 00	9.92E-01	4.06E + 01	2.28E-01	**4.44E-02**
	Rank	5	9	12	10	3	4	11	6	8	7	2	**1**
F6	Ave	6.79E-01	3.64E-01	2.49E + 00	4.51E-04	1.81E-02	2.50E-05	1.08E + 01	1.93E-03	2.07E + 00	**7.46E-07**	6.43E-03	5.46E-06
	Std	4.09E-01	2.04E-01	1.10E + 00	3.05E-04	6.68E-02	1.50E-05	2.74E + 01	1.68E-03	1.28E + 00	**9.71E-07**	3.52E-02	1.04E-05
	Rank	9	8	11	5	6	4	12	7	10	**1**	3	2
F7	Ave	2.12E-03	3.31E-03	1.82E + 01	5.64E-02	1.26E-03	1.76E-03	9.17E-02	4.26E-02	2.85E-04	3.76E-01	5.82E-04	**4.55E-05**
	Std	8.72E-04	3.31E-03	1.45E + 01	1.24E-02	8.68E-04	1.20E-03	5.27E-02	1.77E-02	3.55E-04	1.40E-01	4.76E-04	**4.01E-05**
	Rank	7	6	12	9	4	5	10	8	2	11	3	**1**
F8	Ave	−5.82E + 03	−1.05E + 04	−6.19E + 03	−9.82E + 03	−9.01E + 03	−9.73E + 03	−8.58E + 03	−8.46E + 03	−8.56E + 03	**−1.20E + 04**	−9.24E + 03	−1.19E + 04
	Std	1.07E + 03	1.65E + 03	1.25E + 03	4.29E + 02	2.08E + 03	3.51E + 02	6.87E + 02	1.01E + 03	1.39E + 03	**2.55E + 02**	5.21E + 02	8.34E + 02
	Rank	12	3	11	4	7	5	10	9	8	2	6	**1**
F9	Ave	2.51E + 00	3.79E-15	1.69E + 02	8.40E + 01	2.65E-01	**0.00E + 00**	7.86E + 01	4.47E + 01	**0.00E + 00**	2.02E + 01	4.64E-01	**0.00E + 00**
	Std	3.16E + 00	2.08E-14	2.88E + 01	7.62E + 00	1.45E + 00	**0.00E + 00**	2.19E + 01	2.45E + 01	**0.00E + 00**	5.67E + 00	2.54E + 00	**0.00E + 00**
	Rank	7	4	12	11	5	**1**	10	9	2	8	6	3
F10	Ave	9.96E-14	4.35E-15	2.65E + 00	5.48E-03	**4.44E-16**	5.63E-16	4.55E + 00	2.80E-03	**4.44E-16**	1.83E + 00	6.81E-16	**4.44E-16**
	Std	1.69E-14	2.85E-15	4.72E-01	1.17E-03	**0.00E + 00**	6.49E-16	2.09E + 00	1.61E-03	**0.00E + 00**	1.04E + 00	9.01E-16	**0.00E + 00**
	Rank	7	6	11	9	**1**	4	12	8	2	10	5	3
F11	Ave	5.89E-03	**0.00E + 00**	1.21E-01	6.44E-03	**0.00E + 00**	**0.00E + 00**	1.14E + 00	9.28E-03	**0.00E + 00**	2.07E-02	**0.00E + 00**	**0.00E + 00**
	Std	8.85E-03	**0.00E + 00**	4.53E-02	7.75E-03	**0.00E + 00**	**0.00E + 00**	5.50E-01	1.75E-02	**0.00E + 00**	3.22E-02	**0.00E + 00**	**0.00E + 00**
	Rank	7	**1**	11	9	2	3	12	8	4	10	5	6
F12	Ave	4.83E-02	3.17E-02	4.38E-02	6.55E-05	1.56E-03	6.24E-07	4.58E + 00	6.98E-03	5.96E-02	2.66E + 00	3.46E-03	**3.29E-07**
	Std	3.29E-02	3.45E-02	3.63E-02	4.71E-05	8.25E-03	**3.87E-07**	2.86E + 00	2.63E-02	3.31E-02	2.74E + 00	1.89E-02	8.87E-07
	Rank	9	7	8	5	4	3	12	6	10	11	2	**1**
F13	Ave	6.14E-01	5.22E-01	6.79E-01	2.35E-04	6.20E-01	3.81E-04	1.61E + 01	7.86E-03	1.59E + 00	4.20E-01	6.87E-02	**6.55E-06**
	Std	2.76E-01	2.51E-01	2.69E-01	1.48E-04	4.52E-01	2.01E-03	8.03E + 00	9.34E-03	4.45E-01	9.69E-01	9.10E-02	**1.35E-05**
	Rank	9	8	10	3	7	2	12	5	11	6	4	**1**
F14	Ave	4.95E + 00	2.70E + 00	2.74E + 00	1.20E + 00	1.20E + 00	**9.98E-01**	1.13E + 00	1.10E + 00	1.06E + 00	**9.98E-01**	9.98E-01	1.13E + 00
	Std	4.26E + 00	3.04E + 00	2.13E + 00	9.12E-01	5.47E-01	**0.00E + 00**	4.31E-01	4.00E-01	2.52E-01	**0.00E + 00**	6.51E-16	5.03E-01
	Rank	12	11	10	4	8	**1**	6	3	7	2	5	9
F15	Ave	4.39E-03	8.20E-04	9.05E-04	1.28E-03	7.11E-04	3.08E-04	3.85E-03	**3.07E-04**	1.85E-03	1.75E-03	3.17E-03	**3.07E-04**
	Std	8.12E-03	5.61E-04	1.38E-04	2.90E-03	3.58E-04	2.89E-07	7.52E-03	**4.63E-12**	5.05E-03	5.07E-03	6.87E-03	8.06E-06
	Rank	8	10	12	11	9	3	5	**1**	6	2	7	4
F16	Ave	−1.03E + 00	−1.03E + 00	−1.03E + 00	−1.03E + 00	−1.03E + 00	−1.03E + 00	−1.03E + 00	−1.03E + 00	−1.03E + 00	−1.03E + 00	−1.03E + 00	**−1.03E + 00**
	Std	2.22E-08	1.69E-09	4.61E-16	6.78E-16	5.83E-16	6.52E-16	6.12E-16	6.71E-16	5.98E-16	6.12E-16	6.52E-16	**4.44E-16**
	Rank	12	11	9	**1**	8	3	6	10	7	5	4	2
F17	Ave	3.98E-01	3.98E-01	**3.98E-01**	**3.98E-01**	3.98E-01	**3.98E-01**	3.98E-01	3.98E-01	**3.98E-01**	**3.98E-01**	**3.98E-01**	**3.98E-01**
	Std	2.72E-06	1.21E-05	**0.00E + 00**	**0.00E + 00**	3.24E-16	**0.00E + 00**	4.51E-14	**0.00E + 00**	**0.00E + 00**	**0.00E + 00**	**0.00E + 00**	1.77E-11
	Rank	12	11	**1**	2	8	3	9	10	5	6	7	4
F18	Ave	3.00E + 00	3.00E + 00	3.00E + 00	3.00E + 00	3.90E + 00	3.00E + 00	3.90E + 00	3.00E + 00	3.00E + 00	3.00E + 00	5.70E + 00	**3.00E + 00**
	Std	5.10E-05	1.89E-04	4.28E-15	1.33E-15	4.93E + 00	8.19E-12	4.93E + 00	2.13E-15	1.69E-15	1.26E-15	1.48E + 01	**1.25E-15**
	Rank	12	11	10	3	8	4	5	9	7	**1**	6	2
F19	Ave	−3.86E + 00	−3.86E + 00	**−3.86E + 00**	**−3.86E + 00**	−3.86E + 00	**−3.86E + 00**	**−3.86E + 00**	−3.86E + 00	**−3.86E + 00**	−3.86E + 00	**−3.86E + 00**	**−3.86E + 00**
	Std	2.31E-03	1.01E-02	**1.83E-15**	2.71E-15	3.38E-03	2.68E-15	2.67E-15	2.71E-15	2.33E-15	2.68E-15	2.65E-15	1.27E-08
	Rank	11	12	9	**1**	7	3	5	10	8	4	6	2
F20	Ave	−3.27E + 00	−3.20E + 00	−3.26E + 00	−3.31E + 00	−3.21E + 00	**−3.32E + 00**	−3.28E + 00	−3.32E + 00	−3.28E + 00	−3.29E + 00	−3.28E + 00	−3.30E + 00
	Std	7.42E-02	9.79E-02	6.03E-02	2.99E-02	8.88E-02	4.51E-02	5.83E-02	2.17E-02	7.32E-02	5.54E-02	5.70E-02	**1.53E-12**
	Rank	11	12	9	6	10	2	5	**1**	8	4	3	7
F21	Ave	−9.14E + 00	−8.51E + 00	−7.22E + 00	−9.64E + 00	−6.86E + 00	**−1.02E + 01**	−7.23E + 00	−1.78E-07	−1.02E + 01	−6.48E + 00	−9.47E + 00	−1.02E + 01
	Std	2.34E + 00	2.52E + 00	3.09E + 00	1.52E + 00	2.58E + 00	**1.78E-07**	3.48E + 00	1.28E + 00	4.37E-03	3.58E + 00	1.76E + 00	**1.78E-07**
	Rank	10	12	9	7	11	2	6	**1**	4	8	3	5
F22	Ave	−1.02E + 01	−8.24E + 00	−9.43E + 00	−1.04E + 01	−7.93E + 00	−1.04E + 01	−8.38E + 00	−9.96E + 00	−1.04E + 01	−9.04E + 00	−1.02E + 01	**−1.04E + 01**
	Std	9.63E-01	2.92E + 00	2.32E + 00	6.28E-02	2.68E + 00	**1.21E-07**	3.21E + 00	1.69E + 00	2.70E-06	2.54E + 00	9.70E-01	**1.21E-07**
	Rank	11	12	7	6	10	9	5	**1**	8	4	3	2
F23	Ave	−1.05E + 01	−6.60E + 00	−1.02E + 01	−1.05E + 01	−9.29E + 00	−1.05E + 01	−8.12E + 00	−1.03E + 01	−9.80E + 00	−9.19E + 00	**−1.05E + 01**	**−1.05E + 01**
	Std	9.86E-04	3.64E + 00	1.36E + 00	6.52E-03	2.58E + 00	2.03E-15	3.51E + 00	1.22E + 00	2.25E + 00	2.75E + 00	**1.71E-15**	7.41E-08
	Rank	11	12	8	7	5	10	6	**1**	9	4	2	3
Ave.Rank		9.04	8.65	9.61	6.74	5.83	4.04	8.78	6.30	6.22	6.09	3.96	**2.74**
Friedman		11	9	12	8	4	3	10	7	6	5	2	**1**

As seen in Table 2, MESBOA achieves the highest number of bolded values, which strongly demonstrates the effectiveness of the proposed method in this test suite, highlighting its significant advantage over most of the compared algorithms. Looking at Fig 3, whether for unimodal, multimodal, or hybrid functions, MESBOA’s convergence curve shows a consistently accelerating descent across various test functions. In contrast, algorithms such as GWO, PSO, WOA, DBO, RBMO, and DE tend to become trapped in local optima, making it difficult for them to explore better solutions once they are stuck. Compared to the standard SBOA and other competitive optimizers, MESBOA converges faster and is able to quickly find solutions closer to the optimal. This clearly indicates that the three improvement strategies we adopted are effective, not only helping the algorithm escape local optima but also significantly enhancing its convergence speed and accuracy.

**Fig 4:**
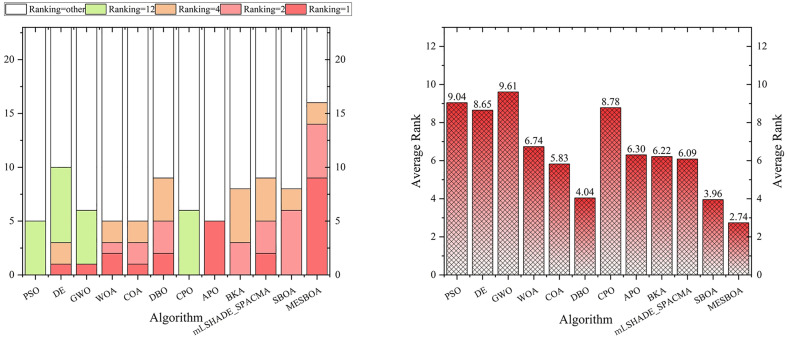
Ranking distribution and average ranking on 23 benchmark functions.

Additionally, to provide a clearer insight into the performance of each algorithm, we present the ranking distribution and average values reported in Table 2 in graphical form, as shown in Fig 4. From Fig 4(a), it is evident that MESBOA ranks first in 9 out of 23 test functions, second in 5 functions, third in 2 function. From the ranking perspective, this strongly highlights the stability of its performance, especially when dealing with unimodal and composite functions, where MESBOA’s performance is exceptional. In contrast, the standard SBOA did not achieve the best ranking in any function, while WOA and DBO only ranked first in 2 test functions, DE and GWO only ranked first in 1 function, and other algorithms did not achieve the best ranking in any function. This further emphasizes the significant improvement MESBOA has achieved over the standard SBOA. Looking at Fig 4(b), which displays the average rankings of each algorithm, MESBOA has an average ranking of 2.74, significantly outperforming DBO with an average ranking of 4.04. GWO performed the worst, with an average ranking of 9.61. The recently proposed CPO and mLSHADE-SPACMA also performed better than other algorithms with an average ranking of 8.78 and 6.09, making it the second-best after MESBOA and SBOA, but still not surpassing the MESBOA proposed in this paper. These results comprehensively confirm the superior performance of the MESBOA algorithm presented in this paper.

### 3.3. Assessing performance with CEC2022 benchmark functions

**Fig 5:**
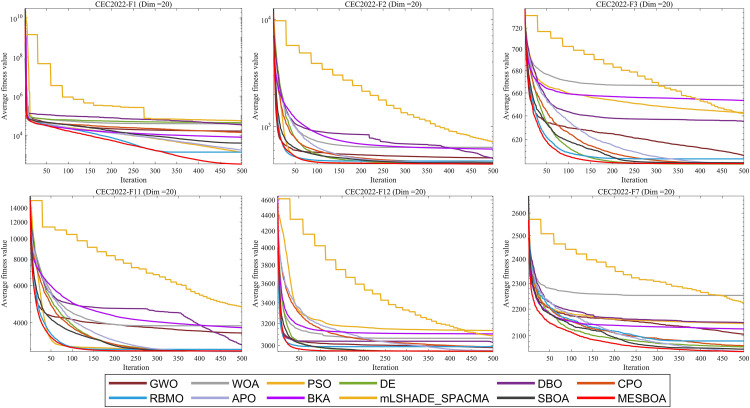
Convergence curves obtained by different algorithms on CEC2022 benchmark functions.

This section employs the challenging CEC2022 benchmark suite for evaluation. The benchmark comprises 12 test functions categorized into four distinct groups: unimodal, multimodal, hybrid, and composition functions. Fig 5 presents the convergence curves of MESBOA on this test suite, while Table 3 provides a comprehensive analysis of the fitness values obtained by each algorithm across 12 independent trials when solving the CEC2022 test functions.

**Table 3:** Results obtained by different algorithms on CEC2022 benchmark functions.

F~	Metric	PSO	DE	GWO	WOA	COA	DBO	CPO	APO	BKA	mLSHADE_SPACMA	SBOA	MESBOA
F1	Ave	1.61E + 04	3.85E + 04	1.32E + 03	4.33E + 04	3.43E + 04	1.37E + 04	1.35E + 03	1.68E + 03	7.83E + 03	5.48E + 04	3.94E + 03	**3.32E + 02**
	Std	4.28E + 03	2.11E + 04	8.49E + 02	9.41E + 03	1.11E + 04	3.32E + 03	1.14E + 03	9.29E + 02	7.72E + 03	1.94E + 04	2.30E + 03	**3.90E + 01**
	Rank	8	10	2	11	9	7	3	4	6	12	5	**1**
F2	Ave	5.10E + 02	6.39E + 02	4.51E + 02	4.52E + 02	5.07E + 02	4.62E + 02	4.76E + 02	4.59E + 02	6.16E + 02	7.25E + 02	4.57E + 02	**4.46E + 02**
	Std	4.10E + 01	9.68E + 01	3.87E + 01	**8.44E + 00**	5.57E + 01	1.37E + 01	2.49E + 01	1.16E + 01	2.81E + 02	9.31E + 01	1.43E + 01	1.78E + 01
	Rank	9	11	3	2	8	6	7	4	10	12	5	**1**
F3	Ave	6.07E + 02	6.67E + 02	6.43E + 02	**6.00E + 02**	6.35E + 02	6.00E + 02	6.04E + 02	6.00E + 02	6.53E + 02	6.42E + 02	6.01E + 02	6.00E + 02
	Std	3.92E + 00	1.19E + 01	9.77E + 00	**2.90E-03**	1.22E + 01	1.18E-01	3.52E + 00	1.97E-01	1.04E + 01	8.30E + 00	1.31E + 00	7.87E-01
	Rank	7	12	10	**1**	8	3	6	4	11	9	5	2
F4	Ave	8.59E + 02	9.46E + 02	8.71E + 02	9.24E + 02	9.15E + 02	8.99E + 02	8.47E + 02	**8.34E + 02**	8.75E + 02	9.75E + 02	8.46E + 02	8.45E + 02
	Std	2.48E + 01	2.34E + 01	1.54E + 01	**7.69E + 00**	2.55E + 01	1.44E + 01	1.12E + 01	1.09E + 01	1.14E + 01	1.86E + 01	1.67E + 01	1.09E + 01
	Rank	5	11	6	10	9	8	4	**1**	7	12	3	2
F5	Ave	1.30E + 03	4.22E + 03	2.39E + 03	1.17E + 03	2.00E + 03	9.13E + 02	1.06E + 03	9.77E + 02	2.23E + 03	2.86E + 03	1.03E + 03	**9.12E + 02**
	Std	3.22E + 02	1.60E + 03	4.34E + 02	1.78E + 02	5.80E + 02	2.18E + 01	1.40E + 02	1.92E + 02	4.11E + 02	1.08E + 03	1.73E + 02	**9.00E + 00**
	Rank	7	12	10	6	8	**1**	5	3	9	11	4	2
F6	Ave	2.32E + 06	4.98E + 06	5.73E + 03	6.34E + 06	4.71E + 05	2.67E + 04	5.84E + 03	4.64E + 03	2.47E + 06	6.52E + 07	6.14E + 03	**4.35E + 03**
	Std	5.55E + 06	5.60E + 06	4.62E + 03	4.25E + 06	2.35E + 06	3.03E + 04	5.56E + 03	**2.74E + 03**	1.30E + 07	4.77E + 07	5.61E + 03	3.96E + 03
	Rank	9	10	5	11	6	8	2	3	7	12	4	**1**
F7	Ave	2.11E + 03	2.25E + 03	2.15E + 03	2.06E + 03	2.15E + 03	2.06E + 03	2.08E + 03	2.04E + 03	2.13E + 03	2.22E + 03	2.05E + 03	**2.04E + 03**
	Std	6.12E + 01	6.95E + 01	6.18E + 01	7.47E + 00	4.89E + 01	9.07E + 00	4.74E + 01	1.25E + 01	3.65E + 01	6.81E + 01	1.29E + 01	**1.27E + 00**
	Rank	7	12	9	4	10	5	6	2	8	11	3	**1**
F8	Ave	2.28E + 03	2.30E + 03	2.31E + 03	2.23E + 03	2.31E + 03	2.23E + 03	2.24E + 03	**2.23E + 03**	2.26E + 03	2.33E + 03	2.23E + 03	2.23E + 03
	Std	6.22E + 01	8.00E + 01	8.65E + 01	2.13E + 00	7.61E + 01	**2.13E + 00**	2.31E + 01	3.01E + 00	5.15E + 01	7.39E + 01	4.96E + 00	2.13E + 00
	Rank	9	11	8	4	10	6	5	**1**	7	12	3	2
F9	Ave	2.53E + 03	2.62E + 03	**2.47E + 03**	2.48E + 03	2.52E + 03	2.48E + 03	2.48E + 03	2.48E + 03	2.52E + 03	2.60E + 03	2.48E + 03	2.48E + 03
	Std	4.12E + 01	6.45E + 01	1.34E-01	9.55E-03	3.68E + 01	3.51E-01	5.31E-01	1.29E + 00	8.58E + 01	4.07E + 01	1.88E-02	**3.61E-07**
	Rank	10	11	**1**	4	8	6	5	7	9	12	3	2
F10	Ave	3.77E + 03	5.14E + 03	4.04E + 03	2.60E + 03	3.67E + 03	2.55E + 03	3.84E + 03	**2.51E + 03**	4.12E + 03	4.06E + 03	2.66E + 03	2.68E + 03
	Std	9.00E + 02	1.24E + 03	9.36E + 02	1.38E + 02	1.21E + 03	1.19E + 02	9.43E + 02	**3.49E + 01**	1.15E + 03	1.74E + 03	3.13E + 02	1.89E + 02
	Rank	8	12	10	5	6	2	7	**1**	11	9	3	4
F11	Ave	3.58E + 03	3.87E + 03	2.92E + 03	2.93E + 03	3.14E + 03	2.94E + 03	2.99E + 03	2.92E + 03	3.79E + 03	4.76E + 03	2.93E + 03	**2.92E + 03**
	Std	3.26E + 02	3.86E + 02	9.49E + 01	8.05E + 01	2.70E + 02	7.58E + 01	1.65E + 02	**4.07E + 01**	7.05E + 02	5.87E + 02	8.05E + 01	1.10E + 02
	Rank	9	11	6	2	8	5	7	3	10	12	4	**1**
F12	Ave	2.98E + 03	3.06E + 03	3.14E + 03	**2.95E + 03**	3.03E + 03	2.99E + 03	2.99E + 03	2.96E + 03	3.10E + 03	3.09E + 03	2.95E + 03	2.95E + 03
	Std	2.82E + 01	8.24E + 01	2.17E + 02	**2.66E + 00**	5.31E + 01	1.19E + 01	3.62E + 01	7.59E + 00	1.07E + 02	3.91E + 01	1.26E + 01	1.09E + 01
	Rank	5	10	8	**1**	9	7	6	4	11	12	3	2
Ave.Rank		7.75	11.08	6.50	5.08	8.25	5.33	5.25	3.08	8.83	11.33	3.75	**1.75**
Friedman		8	11	7	4	9	6	5	2	10	12	3	**1**

As shown in Fig 5, MESBOA demonstrates superior performance compared to other competing algorithms, exhibiting not only faster convergence speed but also an enhanced ability to escape local optima, ultimately achieving higher solution accuracy. The algorithm shows relatively good overall performance in both precisely optimizing unimodal functions and tackling the global search challenges posed by multimodal functions. Although MESBOA may temporarily become trapped in local optima on certain functions (e.g., F6), it consistently demonstrates the capability to escape these suboptimal solutions and continue searching for better ones as iterations progress. These results confirm that MESBOA successfully maintains strong exploratory capabilities while achieving rapid convergence, striking an effective balance between global exploration and local exploitation.

**Fig 6:**
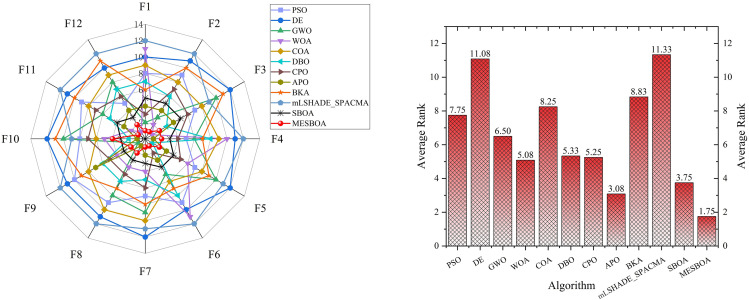
Ranking distribution and average ranking on CEC2022 benchmark functions.

To provide a more intuitive comparison of optimization performance across algorithms, Fig 6 presents the ranking distribution and average rankings from Table 3 in radar chart and bar graph formats. The visualization clearly demonstrates the superior performance of our proposed MESBOA algorithm: it exhibits the smallest radar chart coverage area, with most rankings consistently within the top 4 and no instances of last-place performance. The results indicate that MESBOA performs stably and effectively in all test function categories. Notably, MESBOA achieves an outstanding average ranking of 1.75, significantly outperforming the standard SBOA’s 3.75. This comparison not only highlights MESBOA’s superiority but also provides direct evidence for the effectiveness of our three proposed improvement strategies. The modifications substantially enhance the algorithm’s convergence speed and solution accuracy, establishing MESBOA as a highly competitive optimizer in the field of computational intelligence.

### 3.4. Stability analysis

**Fig 7:**
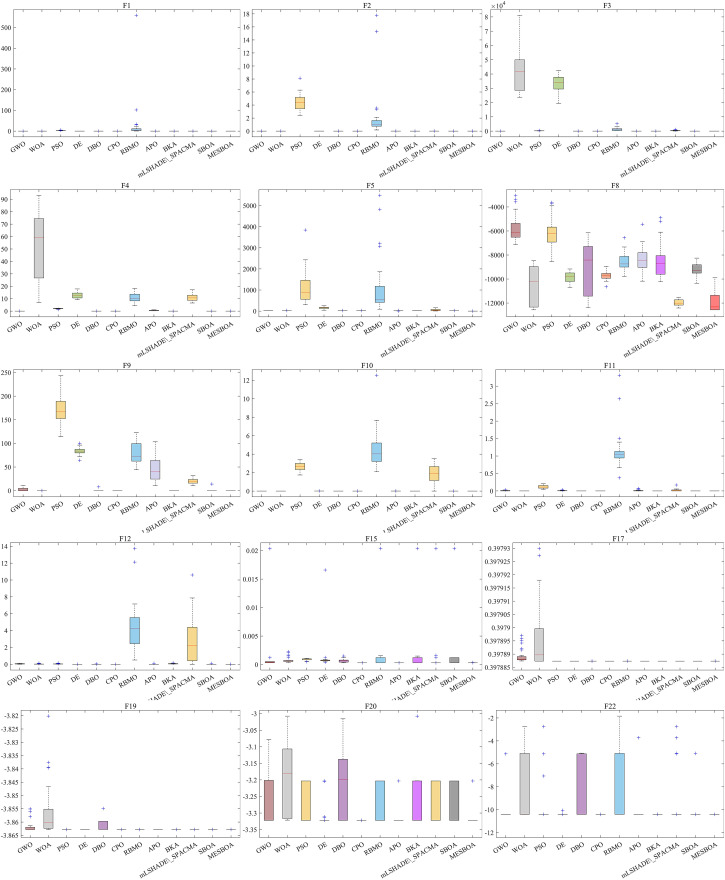
Different algorithms in 23 benchmark functions to obtain partial boxplot.

To more accurately assess the stability of the MESBOA algorithm, we plotted the box plot shown in Fig 7. From the figure, it is clear that, whether dealing with unimodal functions, multimodal functions, or hybrid functions, the data distribution of MESBOA is overall more compact and concentrated. This feature indicates that MESBOA’s search capability is both robust and stable. This is attributed to the three integrated improvement mechanisms within MESBOA, which help the algorithm continuously adjust its search state, effectively mitigating the impact of complex problems on the search process.

Although PSO, DE, CPO, and the standard SBOA also exhibit relatively stable data distributions, their box plots tend to be higher, as seen in functions such as F11, F13, and F15. This phenomenon indirectly reflects that these algorithms may not possess significant advantages and their stability is lacking. In contrast, MESBOA stands out among all algorithms, showcasing the most remarkable stability, as it occupies the lowest position in the box plot. This further validates the effectiveness of the MESBOA algorithm proposed in this paper. This success is attributed to the embedded improvement strategies in SBOA, which not only enhance the algorithm’s solution accuracy but also improve its stability.

### 3.5. Wilcoxon rank statistical test

To comprehensively demonstrate the superiority of the proposed algorithm, this section uses the Wilcoxon rank-sum test to evaluate the performance of MESBOA in each experiment and compares it with other algorithms at a significance level of p = 0.05. The null hypothesis $\left(H_{0}\right)$ is that there is no significant difference between the two algorithms. When the p-value is less than 0.05, the null hypothesis is rejected, indicating a significant difference between the two algorithms. When the p-value is greater than 0.05, the null hypothesis is accepted, suggesting that the performance difference between the two algorithms is not significant, i.e., the algorithms perform similarly. The differences between the algorithms are presented in graphical form, with sections where the p-value is greater than 0.05 highlighted. The test results are shown in Table 4 and Table 5.

**Table 4:** P-value on 23 benchmark functions (dim = 30).

Function	PSO	DE	GWO	WOA	COA	DBO	CPO	APO	BKA	mLSHADE_SPACMA	SBOA
F1	1.21E-12	1.21E-12	1.21E-12	1.21E-12	1.21E-12	1.21E-12	1.21E-12	1.21E-12	1.21E-12	1.21E-12	1.21E-12
F2	3.16E-12	3.16E-12	3.16E-12	3.16E-12	3.16E-12	3.16E-12	3.16E-12	3.16E-12	3.16E-12	3.16E-12	3.16E-12
F3	1.21E-12	1.21E-12	1.21E-12	1.21E-12	1.21E-12	4.57E-12	1.21E-12	1.21E-12	1.21E-12	1.21E-12	1.21E-12
F4	1.72E-12	1.72E-12	1.72E-12	1.72E-12	1.72E-12	1.72E-12	1.72E-12	1.72E-12	1.72E-12	1.72E-12	1.72E-12
F5	3.02E-11	3.02E-11	3.02E-11	3.02E-11	3.02E-11	3.02E-11	3.02E-11	3.02E-11	3.02E-11	3.02E-11	3.02E-11
F6	3.02E-11	3.02E-11	3.02E-11	3.02E-11	5.49E-11	1.07E-07	3.02E-11	3.02E-11	3.02E-11	**4.04E-01**	1.06E-03
F7	3.02E-11	4.98E-11	3.02E-11	3.02E-11	3.02E-11	3.02E-11	3.02E-11	3.02E-11	4.57E-09	3.02E-11	9.92E-11
F8	3.02E-11	8.12E-04	3.02E-11	4.20E-10	2.57E-07	1.09E-10	3.02E-11	3.69E-11	4.98E-11	**1.91E-01**	5.49E-11
F9	1.13E-12	**3.34E-01**	1.21E-12	1.21E-12	**3.34E-01**	3.02E-11	1.21E-12	1.21E-12	4.57E-09	1.21E-12	**3.34E-01**
F10	1.13E-12	1.30E-08	1.21E-12	1.21E-12	2.57E-07	**3.34E-01**	1.21E-12	1.21E-12	3.02E-11	1.21E-12	**1.61E-01**
F11	1.46E-04	3.02E-11	1.21E-12	1.21E-12	6.52E-09	3.57E-06	1.21E-12	1.21E-12	4.57E-09	1.21E-12	2.92E-09
F12	3.02E-11	3.02E-11	3.02E-11	3.02E-11	6.52E-09	3.57E-06	3.02E-11	3.02E-11	3.02E-11	3.02E-11	8.66E-05
F13	3.02E-11	3.02E-11	3.02E-11	3.02E-11	3.02E-11	7.74E-06	3.02E-11	3.02E-11	3.02E-11	1.96E-10	2.92E-09
F14	1.30E-10	1.08E-09	1.98E-03	5.73E-09	**1.95E-01**	8.51E-12	4.90E-05	4.73E-09	4.36E-03	8.51E-12	5.46E-08
F15	4.44E-07	3.34E-11	3.02E-11	3.02E-11	3.26E-07	1.17E-09	**2.01E-01**	2.96E-11	**7.73E-02**	9.40E-06	**9.00E-01**
F16	1.08E-11	1.08E-11	**1.98E-01**	1.74E-11	6.71E-05	5.21E-09	2.77E-06	8.39E-11	1.48E-05	2.77E-06	5.21E-09
F17	3.02E-11	6.72E-10	1.21E-12	1.21E-12	1.72E-12	1.21E-12	2.37E-12	1.21E-12	1.21E-12	1.21E-12	1.21E-12
F18	2.51E-11	2.51E-11	2.30E-02	5.85E-11	2.09E-02	4.68E-09	6.92E-08	9.64E-11	2.39E-05	1.08E-10	1.02E-06
F19	3.02E-11	3.02E-11	1.88E-11	1.21E-12	1.77E-03	2.36E-12	3.15E-12	1.21E-12	1.22E-11	2.36E-12	4.08E-12
F20	1.07E-07	3.50E-09	6.97E-03	2.65E-03	4.50E-02	1.58E-11	9.72E-03	2.68E-11	8.20E-07	7.28E-04	2.62E-03
F21	3.02E-11	3.02E-11	**4.12E-01**	2.75E-03	1.10E-06	2.36E-12	**3.78E-01**	5.30E-09	2.24E-02	**6.62E-01**	6.85E-07
F22	3.02E-11	3.02E-11	9.51E-06	**9.01E-02**	**1.85E-01**	2.36E-12	7.69E-03	4.22E-09	**1.41E-01**	2.95E-04	9.76E-11
F23	3.02E-11	3.02E-11	4.42E-06	1.31E-04	2.65E-02	5.20E-12	3.33E-02	2.20E-10	3.64E-02	5.74E-05	1.40E-11

**Table 5:** P-value on CEC2022 (dim = 20).

Function	PSO	DE	GWO	WOA	COA	DBO	CPO	APO	BKA	mLSHADE_SPACMA	SBOA
F1	3.02E-11	3.02E-11	4.50E-11	3.02E-11	3.02E-11	3.02E-11	4.50E-11	3.34E-11	3.02E-11	3.02E-11	3.02E-11
F2	2.37E-10	3.02E-11	**3.79E-01**	1.53E-05	8.10E-10	3.09E-06	1.47E-07	5.09E-06	4.97E-11	3.02E-11	7.22E-06
F3	8.15E-11	3.02E-11	3.02E-11	**1.76E-01**	3.02E-11	2.05E-03	1.29E-09	3.03E-03	3.02E-11	3.02E-11	2.01E-04
F4	7.96E-03	3.02E-11	3.08E-08	3.02E-11	8.99E-11	6.70E-11	**2.64E-01**	3.18E-03	2.67E-09	3.02E-11	**9.94E-01**
F5	9.83E-08	3.02E-11	4.08E-11	1.87E-07	2.37E-10	**1.12E-01**	1.87E-05	4.36E-02	4.07E-11	3.02E-11	4.08E-05
F6	4.62E-10	3.02E-11	2.61E-02	3.02E-11	4.22E-04	1.17E-09	**6.31E-01**	**1.49E-01**	7.60E-07	3.02E-11	**4.12E-01**
F7	8.35E-08	3.02E-11	7.39E-11	4.74E-06	4.50E-11	8.84E-07	4.42E-06	**7.73E-01**	4.97E-11	3.02E-11	1.17E-02
F8	9.83E-08	2.87E-10	1.39E-06	6.52E-09	3.82E-09	1.43E-08	5.46E-06	4.21E-02	1.85E-08	2.15E-10	**4.38E-01**
F9	3.02E-11	3.02E-11	3.02E-11	3.02E-11	3.02E-11	3.02E-11	3.02E-11	3.02E-11	3.02E-11	3.02E-11	3.02E-11
F10	4.11E-07	2.39E-08	2.78E-07	**7.73E-01**	1.95E-03	**6.31E-01**	1.43E-05	**1.30E-01**	7.22E-06	9.03E-04	**7.62E-01**
F11	1.33E-10	3.02E-11	2.92E-02	**1.49E-01**	2.03E-07	3.92E-02	3.03E-02	**5.19E-02**	6.06E-11	3.02E-11	3.18E-03
F12	7.09E-08	3.34E-11	1.33E-02	**6.84E-01**	3.16E-10	1.78E-10	9.06E-08	5.32E-03	6.70E-11	3.02E-11	**8.77E-01**

As shown in the table, the data marked as highlighted is relatively sparse, and there are fewer significant differences across the three dimensions, indicating that the newly proposed MESBOA demonstrates a clear distinction from the compared metaheuristic algorithms. Therefore, MESBOA exhibits excellent overall performance among various metaheuristic algorithms, suggesting that the three strategies we introduced effectively enhance the algorithm’s convergence speed and solution accuracy, showing significant differences when compared with other algorithms.

### 3.6. Computational cost analysis

**Fig 8:**
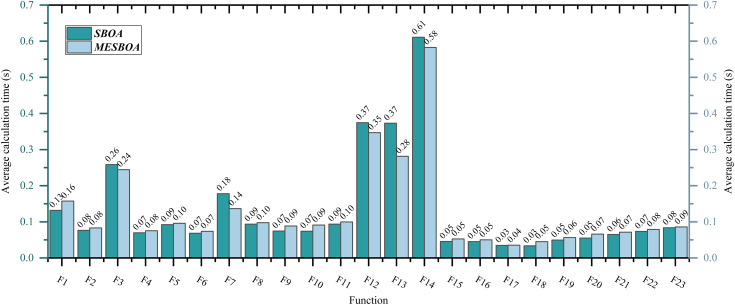
Comparison of computational cost between SBOA and MESBOA.

Based on the previous research findings, it is clear that the optimized MESBOA significantly outperforms the traditional SBOA in terms of overall performance. This section will focus on a more detailed comparative analysis of the computational cost of these two algorithms, with an emphasis on the differences in computational time. To ensure fairness in the comparison, we standardized the parameter settings for both MESBOA and SBOA. In this evaluation, we set the population size to 30 and fixed the maximum number of iterations at 500, with each algorithm running independently 30 times. Fig 8 presents the average computational time (in seconds) for both algorithms when solving each of the test functions.

The experimental results show that, among the 23 benchmark test functions, the execution time of MESBOA and SBOA is similar for single-peak functions and some simple multi-peak functions, with MESBOA performing slightly better than the standard SBOA on F3. Moreover, when handling more complex multi-peak functions (such as F7, F12-F14), MESBOA generally exhibits slightly better computational efficiency. This phenomenon suggests that MESBOA possesses stronger computational capability when dealing with high-complexity problems. Compared to the original SBOA, the improved MESBOA introduces more efficient search mechanisms, enhancing both global search ability and local convergence speed. Overall, MESBOA outperforms the original SBOA in solution accuracy for most test functions, with a negligible increase in runtime, which can almost be ignored.

## 4. Low-light image enhancement based on MESBOA

This chapter focuses on applying MESBOA to low-light image enhancement. First, we present a mathematical model based on the normalized incomplete Beta function for image enhancement. While this function can adapt to various nonlinear transformation curves, conventional parameter determination methods prove inefficient. Therefore, we employ MESBOA to optimize these parameters. Next, we design a fitness function incorporating entropy, edge information, and variance to guide the optimization process. Subsequently, we conduct comprehensive image enhancement experiments on multiple low-light images, comparing the performance of MESBOA and standard SBOA. The evaluation encompasses both visual assessment and quantitative metrics, including Mean Squared Error (MSE), Peak Signal-to-Noise Ratio (PSNR), and Structural Similarity Index (SSIM).

### 4.1. Mathematical model of image enhancement methods

**Fig 9:**
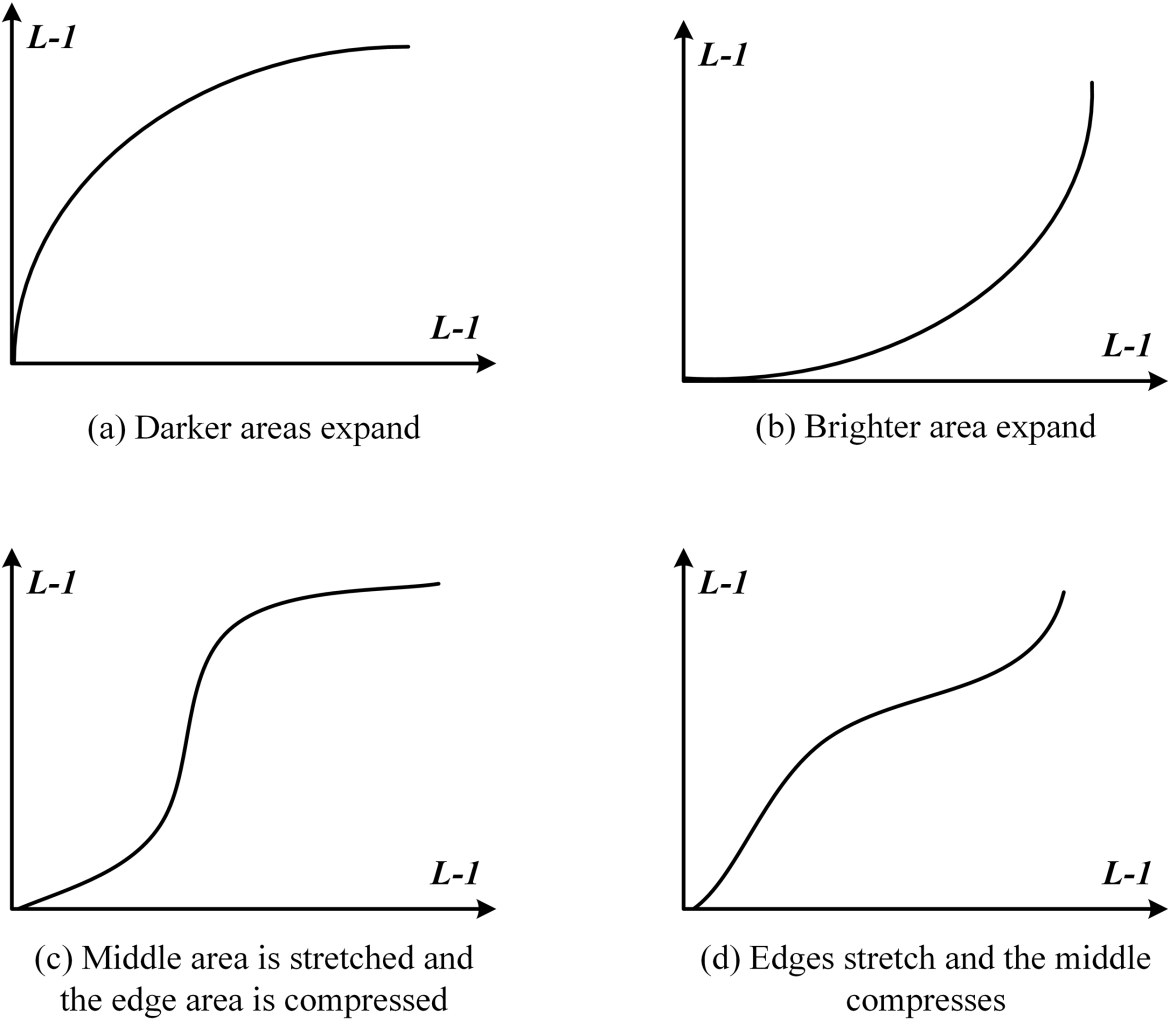
Four typical nonlinear transformation function curves.

For enhancing low-light images, nonlinear transformation functions can be used to adjust the pixel intensity values. Images of varying quality in different environments can be roughly divided into four regions: dark areas, bright areas, edge areas, and central areas. For each of these regions, different nonlinear transformation functions are applied. The specific transformation curves for different regions are shown in Fig 9.

In Fig 9, the horizontal axis represents the grayscale values of the original image, while the vertical axis represents the grayscale values of the image after nonlinear transformation. L-1 represents the grayscale boundary value of the image, typically set as L = 256. As can be seen, depending on the quality of the image, selecting different nonlinear transformations can help enhance the image more effectively. However, the challenge lies in correctly determining which nonlinear transformation function is suitable for each image.

To address the aforementioned issue, Tubbs proposed a normalized incomplete Beta function [60], which can automatically fit the four nonlinear transformation curves based on the image quality. The equation is as follows:


$$\begin{array}{c} \text{F}(u)=B^{-1}(\alpha ,\beta )\times \int_{0}^{u}t^{\alpha -1}(1-t{)}^{\beta -1}dt \end{array}$$ (12)

$$\begin{array}{c} \text{B}(\alpha ,\beta )=\int_{0}^{1}t^{\alpha -1}(1-t{)}^{\beta -1}dt \end{array}$$ (13)

where B(α,β) is the Beta function, 0<(α,β)<10, and u is the original grayscale value of the image. By reasonably adjusting the values of α and β, the incomplete Beta function can fit the four typical nonlinear transformation curves. However, traditional manual adjustment or exhaustive methods to determine α and β are time-consuming, inaccurate, and inefficient. Therefore, MESBOA is combined with the normalized incomplete Beta function, using MESBOA to optimize and search for the best combination of α and β, thereby achieving adaptive image enhancement.

To perform optimization using an optimization algorithm, a fitness function must be defined. It serves as the guiding direction for the algorithm’s progress and directly influences the convergence and stability of the optimization algorithm. In the design of fitness functions for image enhancement, both the overall image and local areas, as well as the balance between different regions, need to be considered. Therefore, to fully reflect various information elements after image enhancement, this chapter’s fitness function design formula includes entropy, edge information, and variance. The specific formula is as follows:


$$\begin{array}{c} fitness=\alpha_{1}\times H+\alpha_{2}\times S+\alpha_{3}\times log(Stv) \end{array}$$ (14)

where H represents the entropy of the image. The higher the entropy value, the greater the amount of information contained in the image. S represents the edge content of the image. The higher the value of, the more edge information the image contains, leading to better contrast. Stv represents the standard deviation of the image’s grayscale values. The larger the variance, the greater the average pixel variation in the image, resulting in better global contrast, which enhances the visibility of the image enhancement effect for the human eye. The coefficients $\alpha_{1},\alpha_{2}\;and\;\alpha_{3}$ are the weighting factors. To make the enhanced image more suitable for human visual observation, these three weighting factors are set to $\alpha_{1}=\frac{1}{4},\alpha_{2}=\frac{1}{4},\alpha_{3}=\frac{3}{4}$.

**Fig 10:**
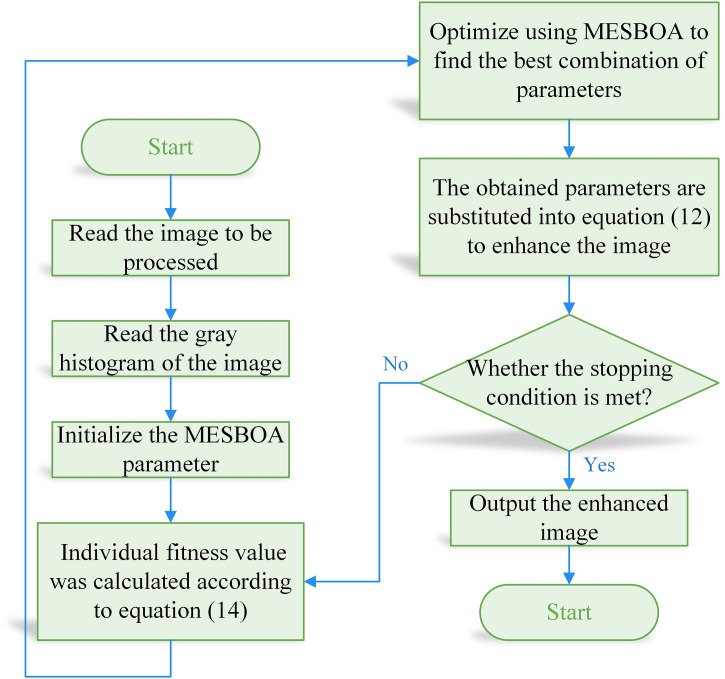
Flowchart of low-light image enhancement based on MESBOA.

**Fig 11:**
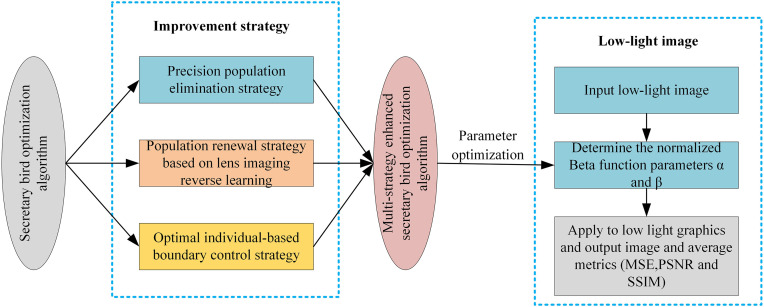
Flowchart of low-light image enhancement based on MESBOA.

The low-light image enhancement process based on MESBOA is shown in Fig 10 and Fig 11.

### 4.2. Experiments and analysis of image enhancement

**Fig 12:**
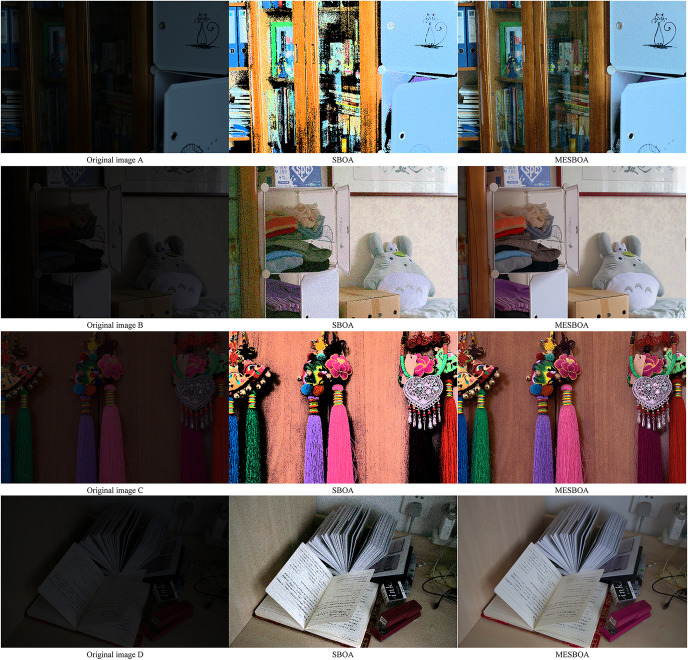
Results of low-light image enhancement test.

To further demonstrate the practicality of MESBOA, we applied it to low-light image enhancement. The low-light image data used in this study was sourced from the publicly available LOL dataset (https://datasets.activeloop.ai/docs/ml/datasets/lol-dataset/). Four low-light images were selected from the internet, all captured at night or under insufficient lighting conditions. In these images, certain object contours and details are difficult to discern with the naked eye. Both MESBOA and SBOA algorithms were used to enhance these images, and the effectiveness of enhancement was evaluated visually. Additionally, the mean squared error (MSE), peak signal-to-noise ratio (PSNR), and structural similarity index (SSIM) of the enhanced images were recorded for both MESBOA and SBOA to quantitatively assess the image enhancement quality, as shown in Table 6. The original images, along with the images enhanced by MESBOA and SBOA, are presented in Fig 12.

As shown in Table 6, MESBOA consistently achieves the lowest MSE across all four images, indicating that the enhanced images have smaller pixel-level differences from the original images. This suggests that the enhanced images are closer to the original ones, resulting in higher image quality. For the second evaluation metric, PSNR, MESBOA also outperforms SBOA in all four images, demonstrating that the noise level relative to the signal strength is lower. This implies that the enhanced images exhibit higher quality and are closer to the original high-quality images, which is generally perceptible to the human eye. Furthermore, in terms of SSIM, MESBOA achieves higher values than SBOA across all four images, approaching 1 more closely. This indicates that MESBOA-enhanced images are structurally more similar to the original ones, further confirming superior image quality.

Furthermore, as observed in Fig 12 MESBOA demonstrates a more visually noticeable enhancement effect on low-light images compared to SBOA. The overall brightness of the images enhanced by MESBOA is higher, and the details of objects in previously dark regions become significantly clearer. Specifically, images enhanced by SBOA exhibit less refined detail processing, appearing somewhat blurry, whereas MESBOA produces images that look more natural and closer to real-world scenes, as evident in Fig 12 (A and C). This further validates the effectiveness of the three improvement strategies embedded in SBOA, which enhance the algorithm’s performance.

**Table 6:** Evaluation metrics obtained by SBOA and MESBOA.

Image	A		B		C		D	
Algorithm	SBOA	MESBOA	SBOA	MESBOA	SBOA	MESBOA	SBOA	MESBOA
MSE	0.2843	**0.2091**	0.3810	**0.3738**	0.5751	**0.3201**	0.2062	**0.2023**
PSNR	5.9924	**6.7955**	3.7090	**3.8490**	4.9467	**6.8738**	6.8580	**6.9410**
SSIM	0.4854	**0.5433**	0.3961	**0.5569**	0.3464	**0.6125**	0.4370	**0.5373**

## 5. Summary and prospect

This paper proposes a Multi-Strategy Enhanced Secretary Bird Optimization Algorithm (MESBOA) to improve the performance of the standard SBOA through a precise population elimination mechanism, a lens-imaging-based reverse learning strategy, and an optimal-individual-based boundary control strategy. Experimental results on 23 benchmark test functions demonstrate that MESBOA achieves faster convergence, higher accuracy, and greater stability with negligible additional computational cost.

As a case study, MESBOA was applied to low-light image enhancement by optimizing the parameters of a normalized incomplete Beta function. The enhanced images exhibit improved objective metrics (MSE, PSNR, and SSIM) and present visually clearer and more natural results. While this application highlights the potential of MESBOA in image enhancement tasks, we acknowledge that the current experiments are primarily illustrative, focusing on demonstrating the algorithm’s optimization capabilities rather than providing a comprehensive comparison with state-of-the-art image enhancement methods.

Although the MESBOA algorithm demonstrates excellent performance in numerical optimization problems and low-light image enhancement tasks, there are still several areas for improvement. Firstly, while the algorithm outperforms others in terms of coverage and optimization results, its convergence speed in the early stages for certain functions (such as F6, F12, etc.) is relatively slow. This may affect efficiency in real-world applications that require fast responses. Secondly, although MESBOA shows strong robustness, its performance stability under large-scale problems or more complex constraint conditions has not been fully validated. Additionally, the sensitivity of the algorithm’s parameters and its adaptability to different types of problems still require further investigation.

Future research can explore several directions to extend the value of MESBOA. First, its applicability could be further validated in critical real-world tasks such as medical image processing (e.g., enhancing low-contrast X-rays or MRI for improved diagnosis) and remote sensing image analysis (e.g., denoising or super-resolution of satellite imagery), which would demonstrate its robustness across domains. Second, combining MESBOA with advanced techniques like deep learning (e.g., hybrid CNN architectures) may synergize their strengths for higher performance. Third, adaptive parameter optimization could be developed to automatically adjust settings for different image types (medical vs. optical), while fourth, reducing computational complexity would enable real-time processing of large-scale data, such as streaming remote sensing datasets. These efforts would collectively expand the algorithm’s practical impact.
